# Short-term efficacy and safety of infliximab biosimilar (HS626) in inducing remission of inflammatory bowel disease: A real-world single-center study

**DOI:** 10.1097/MD.0000000000048853

**Published:** 2026-05-15

**Authors:** Yun-si Huang, Dong-ping Lai, Hui Nong, Tao Zhang, Zi-yao Chen, Wen-bin Zeng, Xu-ping Huang, Yu-yuan Lin

**Affiliations:** a Guangxi University of Chinese Medicine, Nanning, China; b The Second Affiliated Hospital of Guangxi University of Chinese Medicine, Nanning, China; c Wuxi Traditional Chinese Medicine Hospital, Wuxi, China.

**Keywords:** biosimilar, effectiveness study, HS626, inflammatory bowel disease, infliximab, real-world study, safety

## Abstract

HS626, a newly approved infliximab biosimilar in China (2021), initially lacked approval for treating inflammatory bowel disease (IBD). This study aimed to evaluate the short-term efficacy and safety of HS626 in IBD inducing remission. A retrospective study (Jan 2022–Apr 2024) evaluated HS626 in Crohn’s disease (CD) and ulcerative colitis (UC). Disease activity was assessed using the Crohn’s disease activity index and simplified endoscopic score for Crohn’s disease (SES-CD) in CD patients, and the ulcerative colitis endoscopic index of severity, Mayo score, and endoscopic severity in UC patients. Blood samples were collected at weeks 0, 2, 6, and 14 to measure hemoglobin, leukocytes, platelets, albumin, C-reactive protein, and red blood cell count. Paired *t*-tests/Wilcoxon tests were used. Adverse reactions were assessed. In this study, 24 patients (11 UC, 13 CD) were enrolled, 21 patients (10 UC, 11 CD) with endoscopy data at weeks 0, 14 were enrolled. Clinical response rates were 81.8%(9/11) in UC and 69.2%(9/13) in CD. Clinical remission rates were 9.1%(1/11) in UC and 76.9% (10/13) in CD. Atopic dermatitis occurred in 1 patient (sole adverse reaction). HS626 effectively induces remission in IBD patients with a favorable short-term safety profile.

## 1. Introduction

Inflammatory bowel disease (IBD) is a chronic autoimmune disease with nonspecific characteristics, including ulcerative colitis (UC) and Crohn’s disease (CD). IBD prevalence continues to rise worldwide, imposing a considerable financial strain on both patients and society.^[1]^

IBD pathogenesis is associated with an excessive immune response and inflammation. TNF-α is a key pro-inflammatory cytokine that is implicated in the pathogenesis of IBD.^[2]^ Infliximab (IFX) has been identified as an efficacious treatment option for IBD. The effectiveness and safety of subjects treated with infliximab have been assessed in a number of studies.^[3-6]^

Although the efficacy of IFX has been demonstrated, its high cost is unaffordable for some patients. Therefore, the introduction of biosimilar provides a beneficial and low-risk treatment alternative for IBD patients. Biosimilar is biotherapeutic products that exhibit similarity to licensed bio therapeutic products regarding quality, safety, and efficacy. A series of large-scale observational cohort studies on IBD have shown that infliximab biosimilar have comparable effectiveness and safety to the original drug^.[7,8]^ In the case of equal efficacy, biosimilar have a lower cost, which can increase their use.

Infliximab (HS626) is a biosimilar of infliximab (Remicade), used to treat CD in both adults and children over 6 years old, fistulizing CD, and ulcerative colitis in adults. It was launched in China in 2021, making it the second infliximab biosimilar to be approved for marketing in China.

Many studies have confirmed the efficacy of widely used biosimilars, however, clinical data on efficacy and safety in induction of IBD with HS626 (including symptomatic response, symptomatic remission, and normalization of C-reactive protein (CRP) ) are currently lacking. The aim of this study was to thoroughly assess the clinical effectiveness and safety of HS626 in UC and CD patients.

## 2. Materials and methods

### 2.1. Study subjects

This retrospective study was conducted at Ruikang Hospital Affiliated to Chinese Medicine Guangxi University. IBD patients treated with HS626 between January 2022 and April 2024 were enrolled. This study was conducted under Helsinki Ethics for Medical Research, and was approved by Medical Ethics Committee of Ruikang Hospital Affiliated to Guangxi University of Chinese Medicine(KY2024-003). Waiver of informed consent was granted due to retrospective analysis of anonymized data per ethics committee guidelines.

### 2.2. Inclusion criteria

The eligibility criteria were: those who were diagnosed with IBD in accordance with the existing guidance^[9,10]^; patients aged ≥10 years and ≤70 years; patients who underwent endoscopic examination during hospitalization; and those receiving HS626 for at least 14 weeks unless discontinued due to adverse reactions.

### 2.3. Exclusion criteria

The criteria for exclusion were: serious organ disorders of the gastrointestinal system, such as local intestinal stenosis, gut obstruction, bowel perforation, toxic megacolon, or colon cancer; individuals with severe damage to important organs, such as the heart, liver, or kidneys; and pregnancy or lactation.

**Figure 1.**
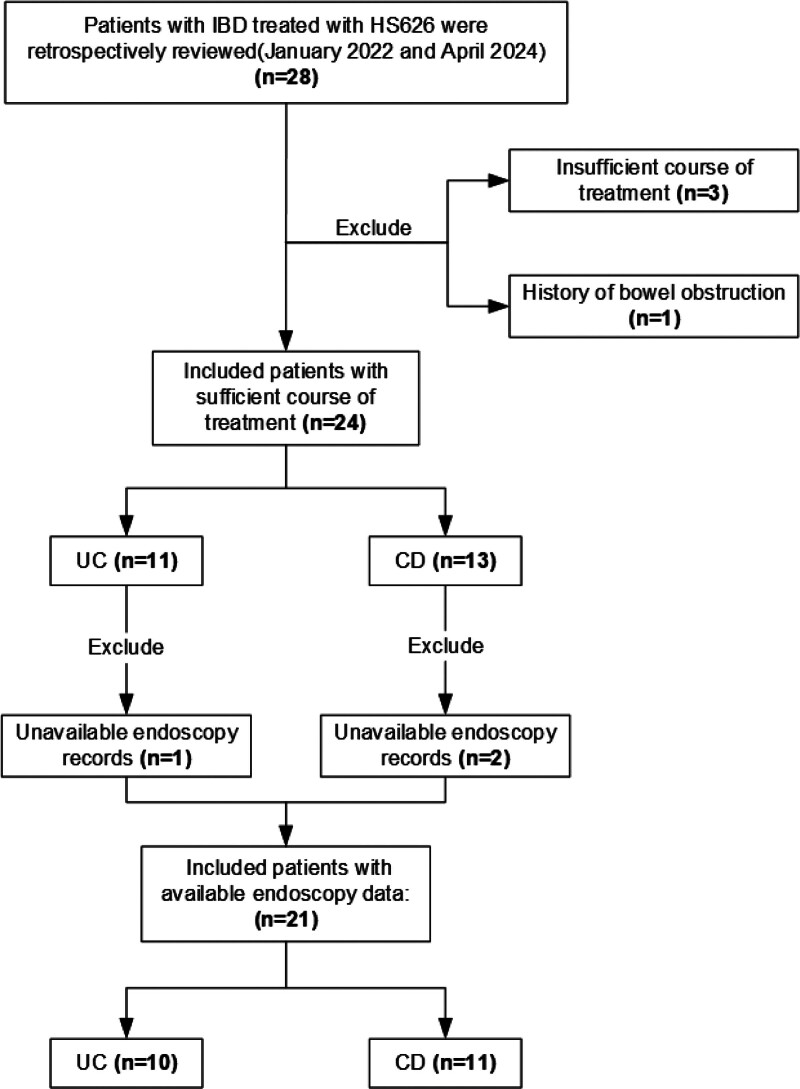
Patient’s procedure chart. In total, 28 subjects received HS626 during this time, finally 24 patients with sufficient course of treatment and 21 patients with endoscopy data at weeks 0,14 were enrolled in the study. Data have been collected in these patients. CD = Crohn’s disease, IBD = inflammatory bowel disease, UC = ulcerative colitis.

Eligible patients were included according to defined eligibility and exclusion criteria (Fig. 1). The sample size of 24 was considered adequate for this single-center retrospective study, as it detected a statistically significant effect (*P* < .01) with a large effect size, indicating sufficient power to address the research objectives.^[11]^ Despite the modest size, complete endoscopic follow-up (week 0 and 14) was available for 87.5% (21/24) of patients, and all laboratory parameters were serially measured at 0, 2, 6, 14 weeks.

### 2.4. Treatment method

HS626 was initially administered at 5 mg/kg on weeks 0, 2, and 6, thereafter, starting with week 14, the patient continued with maintenance treatment every 8 weeks.

### 2.5. Outcomes and definitions

This study assessed the clinical efficacy of HS626 by monitoring changes in clinical signs, including blood cell count, white blood cell count, platelet count, albuminuria, CRP, red blood cell count. The findings of the endoscopy were also considered, and a prospective monitoring of adverse events was performed for the purpose of the safety analysis. In order to assess the effectiveness of HS626, we compared the pre-therapy score with the duration and the degree of remission of the post-therapy. The aim of this study is to establish the efficacy of HS626.

The ulcerative colitis clinical effectiveness was evaluated by Mayo Improvement Score^[12]^ as the standard. The Mayo score ranges from 0 to 12, with higher scores indicating more severe disease. Clinical remission is defined as a score ≤2 with no subscore >1. Clinical response is defined as a decrease from baseline of ≥3 points and ≥30%, plus a decrease in the rectal bleeding subscore of ≥1 or an absolute subscore of 0 or 1. The ulcerative colitis endoscopic index of severity (UCEIS) score ranges from 0 to 8, with higher scores indicating more severe endoscopic activity.^[13]^ UCEIS was 0 to 1 for mucosal healing, 1 to 3 for moderate activity, 4 to 6 for moderate activity, and 7 to 8 for moderate activity.

Clinical efficacy assessments of CD were performed on the basis of changes in the Crohn’s disease activity index (CDAI)^[14]^: clinical remission has been defined as CDAI < 150 points; CDAI ≥ 150 points indicated disease activity, with 150 to 220 points indicate mild, 221 to 450 points indicate moderate, and >450 points indicate severe; the clinical response was defined as CDAI reduction of 100 or more at week 0. 4 clinical nonresponses were defined as CDAI > 150, with an increase of over 100 from week 0. The severity of endoscopic lesions was assessed using the simplified endoscopic score for Crohn’s disease (SES-CD) scoring system^.[15]^

SES-CD scores from 0 to 3 are indicative of mucosal healing, 4 to 10 is intermediate, 11 to 19 is intermediate, and ≥20 is severe endoscopic activity.

The UC Mayo score and CDAI CD optimal score were used in this situation to measure the difference in clinical response from clinical efficacy. The UCEIS score and SES-CD score were defined as treatment effectiveness of HS626 for remission. Clinical efficacy was assessed at weeks 0, 2, 6, and 14.

### 2.6. Statistical analysis

Data were analyzed by SPSS 25. Normal continuity data were assessed by Kolmogorov–Smirnov method. Normally distributed data are expressed as $\bar{x}\pm s$.

For normal distribution data, paired *t*-tests were used, whereas for biased data, the Wilcoxon rank sum test was used, represented by M(IQR) (Median (interquartile range)). As for categorical data, they are presented in proportion and percentage terms, and repeated-measures ANOVA was employed for multiple group comparisons. Laboratory results at baseline, weeks 2, 6, and 14 (including hemoglobin, leukocyte, platelet, albumin, C-reactive protein, and erythrocyte sedimentation rate [ESR]), clinical results at the beginning of the study and the 14th week, as well as the endoscopic results of the 2 groups. The statistical meaning was determined to be *P* < .05.

## 3. Results

### 3.1. Patients’ characteristics

From January 2022 through April 2024, 24 IBD patients in Guangxi University of Chinese Medicine Affiliated Hospital were treated with HS626 and qualified for inclusion. Of them, 13 cases diagnosed with CD, 54.2 percent of the total. 11 cases diagnosed with UC, 45.8 percent of the total. IBD was classified according to the Montreal classification system,^[16]^ which determines the scope of endoscopic lesions. All included participants were inpatients (Table 1).

**Table 1:** Baseline characteristics of patients.

	CD (n = 13)	UC (n = 11)
Sex, n		
Male/female	10/3	4/7
Age (yr), M(IQR)	23 (17, 45.5)	56 (33, 59)
BMI (kg/m^2^), mean (SD)	19.75 ± 10.8	20.3 ± 6.78
Duration of the disease (mo), M(IQR)	20 (6, 71.25)	36 (15, 156)
Previous conventional medications, n(%)		
Mesalazine	8 (61.5%)	10 (90.9%)
Azathioprine	1 (7.7%)	0
Sulfasalazine	0	1 (9.1%)
Methylprednisolone	0	1 (9.1%)
Previous biosimilar agents, n(%)		
Vedolizumab	0	7 (63.6%)
Ustekinumab	2 (15.4%)	0
Infliximab (Remicade)	3 (23.1%)	1 (9.1%)
Disease severity, n(%)		
Mild	2 (15.4%)	0
Moderate	10 (76.9%)	6 (54.5%)
Severe	1 (7.7%)	5 (45.5%)
Behavior of disease (CD cohort only)		
B1	8 (61.5%)	
B2	4 (30.8%)	
B3	1 (7.7%)	
Age at onset in years (CD cohort only)		
A1	2 (15.4%)	
A2	8 (61.5%)	
A3	3 (23.1%)	
Location (CD cohort only)		
L1	6 (46.2%)	
L2	3 (23.1%)	
L3	4 (30.8%)	
L4	0	
Extent of disease (UC cohort only)		
E1		0
E2		6 (54.5%)
E3		5 (45.5%)

E1: rectal involvement; E2: left-sided colitis; E3: extensive colitis.

A1: ≤16 years; A2: 17 to 40 ; A3: >40 years.

L1: terminal ileum; L2: colon; L3: ileum colon; L4: upper gastrointestinal tract.

B1: non-narrow, non-fistula; B2: narrow; B3: fistula.

BMI = body mass index, CD = Crohn’s disease, M(IQR) = median (interquartile range), SD = standard deviation, UC = ulcerative colitis.

### 3.2. Clinical effectiveness

**Figure 2.**
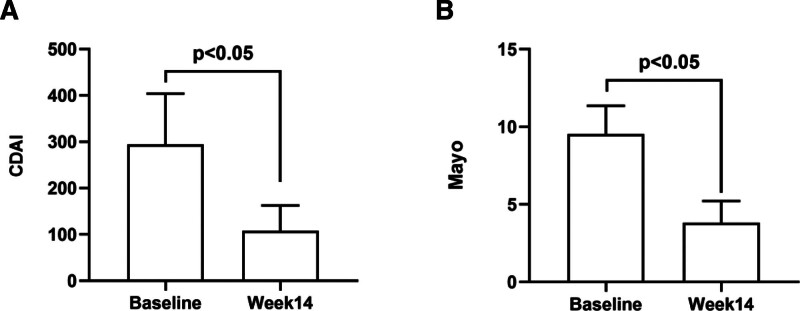
The CDAI scores were evaluated in IBD at the beginning and the end of the 14th week. (A, B) The Mayo scores were assessed for UC patients at the same period. CDAI = Crohn’s disease activity index, IBD = inflammatory bowel disease, UC = ulcerative colitis.

The average CDAI and modified Mayo scores for CD and UC were 294.4 ± 109.5 and 9.5 ± 1.8 at baseline, respectively. After a 14-week treatment with HS626, significant reductions in average scores were observed for both CD and UC patients, and the scores decreased to 108.3 ± 54.5 and 3.8 ± 1.4, respectively (*P* < .05; Fig. 2). Specifically, the clinical remission rate for patients with CD was 76.9% (10/13) after treatment, whereas the clinical response rate was 69.2% (9/13). In comparison, the clinical remission rate in UC patients reached 9 %(1/11), and the clinical response rate was 81.8% (9/11).

### 3.3. Laboratory index evaluation

**Figure 3.**
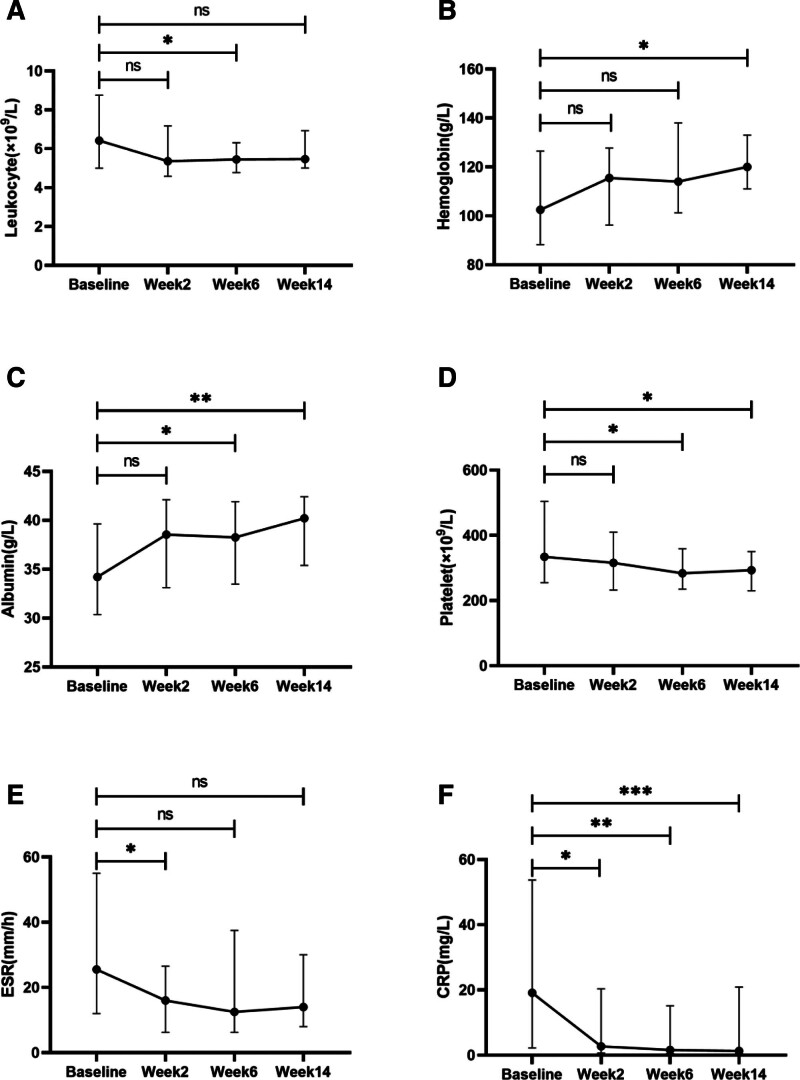
Changes in the IBD patient index were assessed at baseline and 2, 6, and 14 weeks after treatment.(A) leukocytes; (B) hemoglobin; (C) albumin; (D) platelet; (E) ESR; and (F) CRP; data represent M(IQR), Statistical significance: *P < .05, **P < .01, ***P < .001, ns for *P* > .05 (repeated-measures). CRP = C-reactive protein, ESR = erythrocyte sedimentation rate, IBD = inflammatory bowel disease, M(IQR) = Median (interquartile range).

We calculated the M(IQR) for each indicator at baseline and 2 weeks, 6 weeks, and 14 weeks after treatment. Significant reductions in white blood cell count, ESR, and CRP were observed (*P* < .05), alongside increases in hemoglobin and albumin levels compared to baseline. (Fig. 3).

### 3.4. Endoscopic mucosal healing situation

**Figure 4.**
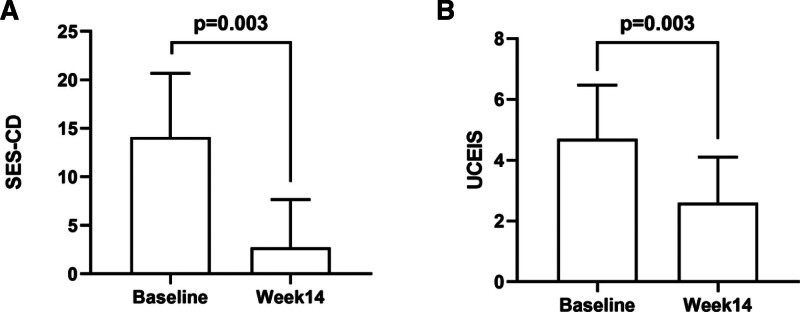
Trends in IBD endoscopic scores were assessed at the start of the study and at the end of 14 weeks. (A) The SES-CD values were assessed in CD patients at the beginning and at the end of the 14th week. (B) The UCEIS values were assessed in UC patients at the beginning and ending of the treatment. Data are shown as average ± SD. Paired *t*-test is used to analyze data, with a statistical significance of <0.05. CD = Crohn’s disease, IBD = inflammatory bowel disease, SD = standard deviation, SES-CD = simplified endoscopic score for Crohn’s disease, UC = ulcerative colitis, UCEIS = ulcerative colitis endoscopic index of severity.

Colonoscopic examination was carried out prior to and at the end of 14 weeks. Mucosal healing rates were 20% (2/10) in UC patients and 81.8% (9/11) in CD patients. The average scores for UCEIS and SES-CD before treating UC and CD patients were 4.7 ± 1.77 and 14.09 ± 6.58, respectively. Compared to before treatment, the average endoscopic scores significantly decreased after treatment (UCEIS ($\bar{x}\pm s$) = 2.6 ± 1.5, SES-CD ($\bar{x}\pm s$) = 2.72 ± 4.92; *P* < .05; Fig. 4).

Among the 24 patients, only 1 experienced adverse reaction during treatment. In the 6th week, the patient developed urticaria, pruritus, and tongue edema after receiving HS626, and was diagnosed with allergic dermatitis. No other severe adverse reactions were observed.

## 4. Discussion

In this study, reviewed clinical data from 24 IBD patients at a single-center over 14 weeks to evaluate the effectiveness and safety of HS626 in inducing IBD remission.

Following 14 weeks of therapy with HS626, we have shown that 24 IBD patients have significantly improved their clinical signs. In addition, the levels of inflammation (ESR, CRP), WBC, and the PLT were lower than those measured at baseline. These changes indicated a reduction in inflammation and disease activity following the 14-week treatment. Furthermore, increased Hb and Alb levels suggest an improved overall nutritional status of patients after treatment. The clinical remission rates of UC patients is 9.1% (1/11), and the clinical remission rates of CD patients are 76.9% (10/13), with clinical response rates of 69.2% (9/13) and 81.8% (9/11), respectively. Although only 1 UC patient (9.1%) met strict remission criteria (Mayo ≤ 2), 81.8% (9/11) achieved clinical response (≥3-point decrease), suggesting meaningful symptom control. Compared with previous research results, these results show similarities on the treatment of IBD using innovative IFX drugs (such as Remicade) and other IFX biosimilar. A previous study demonstrated that the efficacy and safety of infliximab biosimilar in inducing remission in IBD are similar, compared to the reference drug infliximab. The biosimilar arm demonstrated a clinical response rate of 84.2% in CD and 68.4% in UC patients, while that of UC was 81.2% and 68.7% respectively.^[17]^ In another study, 65.5% of CD patients and 75.5% UC patients after 14 weeks of treatment with IFX were achieved. In a retrospective Korean study, the clinical response rate and clinical remission rates in CD and infliximab-treated patients were 94% and 78%, respectively.^[18]^ In a prospective, multicenter, national cohort study in Hungary, biosimilar therapy resulted in an 86% and 49% reduction in the CD group compared with 74% and 56% in the UC group^.[19]^ HS626 demonstrated comparable efficacy to originator infliximab and other infliximab biosimilars in inducing remission, with clinical response rates of 69.2% to 81.8% versus 68.4% to 84.2% in reference biosimilars.^[17,19]^ In our study, 75 percent of CD cases were categorized as moderate or moderately severe, suggesting that biologic therapy may improve remission rates in refractory IBD.

Mucosal healing is an indicator of prognosis for IBD, and it has been linked to lower rates of colectomies, clinical recurrences, and the possibility of dysplasia and colorectal cancer^.[20-23]^ In terms of MH endoscopic examination, UCEIS and SES-CD results showed that UC and CD had a mucosal healing rate of 20% (2/10) and 81.8% (9/11), respectively. The mean endoscopic score after 14 weeks of treatment was significantly lower than that at baseline. Vascular patterns, mucosal bleeding, ulceration, and erosion also showed marked improvements.

Although previous studies have shown that the active substance is identical to that of the original and other biologics, previous studies have shown a similar effect. Bertini et al reported a 60% improvement in mucosal healing in IFX naive CD patients.^[24]^

Nakagawa et al reported that only 29.5 percent of the IBD patients had an endoscopic remission rates score that was “normal” or “mild,” but the mucosal healing rate significantly increased to 60.0% with CT-P13 therapy.^[25]^ Therefore, it can be inferred that HS626 may effectively promote the healing effect of mucosa. Moreover, the influence of HS626 in this respect in UCC patients was superior to that of UC, possibly attributed to restricted sample volume of this study, variations in the scoring criteria, and the presence of some errors. Thus, it is necessary to collect a larger sample size to validate previous findings. The tolerability and safety of HS626, only 1 case (4%) of adverse events was observed after treatment with HS626 in this study, leading to the discontinuation of HS626 in the 6th week of treatment. No unexpected safety outcomes, including death or malignancy, were observed. The incidence of adverse reactions leading to HS626 discontinuation was lower in our study than in previous reference drug studies. Referring to Gecse et al, after 14 weeks of CT-P13 treatment, 7.1% of IBD patients had adverse reactions, 6.6% IBD patients had adverse reactions and 5.7% IBD patients had infectious adverse reactions.^[26]^ According to the research results of Kumaret and others, 24.1% of patients experienced adverse reactions. In their study, 18 patients were diagnosed with tuberculosis (TB), among whom 17 received the original research biosimilar drug and 1 case received a biosimilar.^[17]^ Guerra Veloz et al indicated that the adverse event rate of originator infliximab was 9.2%, and that of the infliximab biosimilar was 11.2%.^[27]^ Our results indicate that patients with CD and UC typically tolerate short-term treatment with HS626, and the adverse event incidence with HS626 (4.2%) was numerically lower than rates reported for CT-P13 (7.1%)^[26]^ and originator infliximab (9.2%),^[27]^ though statistical comparison was precluded by sample size limitations.

This study has some limitations that should be considered: firstly, the research is a single center, and the modest sample size (n = 24) is limited. however, as a real-world study, its strength lies in providing the first efficacy and safety profile of HS626 in Chinese IBD patients, with high data completeness (87.5% endoscopic follow-up). Future multicenter studies with larger studys are needed. Multicenter, prospective studies can evaluate these results more accurately. Secondly, this study focused only on the induced remission effect of HS626 on IBD, and the long-term efficacy of HS626 and its role in maintaining remission were not studied. Last, while the low mucosal healing in UC (20%) limits firm conclusions, it aligns with real-world biosimilar data in biologic-exposed cohorts.^[25]^ Future studies should stratify UC patients by prior biologic exposure to clarify HS626’s efficacy in biologic-naive subgroups. Therefore, Future research should investigate the long-term effects of HS626 in order to provide a more solid basis for clinical practice.

## 5. Conclusion

HS626, as a biosimilar of IFX, showed good efficacy in inducing remission of IBD. It significantly ameliorated patients’ symptoms and inflammatory response within 14 weeks. It also effectively promotes mucosal healing. This rapid therapeutic effect is of great significance in improving patients’ quality of life and controlling disease progression. Meanwhile, the lower side effects suggest that HS626 is safe and reliable. Once again, it has been demonstrated that HS626 showed promising induction efficacy in this preliminary study. In this preliminary retrospective study, HS626 induced clinical remission in 76.9% of CD and 9.1% of UC patients over 14 weeks, acceptable short-term safety. Further randomized studies must validate these findings and explore HS626’s role in maintenance therapy.

## Author contributions

**Conceptualization:** Hui Nong.

**Data curation:** Dong-ping Lai.

**Formal analysis:** Yun-si Huang.

**Investigation:** Dong-ping Lai, Zi-yao Chen, Wen-bin Zeng, Xu-ping Huang, Yu-yuan Lin.

**Methodology:** Yun-si Huang.

**Resources:** Dong-ping Lai, Zi-yao Chen, Wen-bin Zeng, Xu-ping Huang, Yu-yuan Lin.

**Software:** Yun-si Huang.

**Supervision:** Hui Nong, Tao Zhang.

**Validation:** Yun-si Huang.

**Visualization:** Yun-si Huang.

**Writing – original draft:** Yun-si Huang.

**Writing – review & editing:** Yun-si Huang, Hui Nong, Tao Zhang.

## References

[1] Rahmati L Mooghali A Kamani SMH Zare F Askari H Safarpour AR. Economic burden of inflammatory bowel disease in Shiraz, Iran. Arch Iran Med. 2023;26:23–8. 37543918 10.34172/aim.2023.04 PMC10685812

[2] Lu Q Yang MF Liang YJ. Immunology of inflammatory bowel disease: molecular mechanisms and therapeutics. J Inflamm Res. 2022;15:1825–44. 35310454 10.2147/JIR.S353038 PMC8928114

[3] Jeffrey A. Safety and efficacy of transitioning inflammatory bowel disease patients from intravenous to subcutaneous infliximab: a single-center real-world experience. Ann Gastroenterol. 2023;36:549–54. 37664232 10.20524/aog.2023.0816 PMC10433247

[4] Patel S Walsh J Pinnell D. Real-world experience with biosimilar infliximab-adba and infliximab-dyyb among infliximab-naïve patients with inflammatory bowel disease in the Veterans Health Administration. Medicine (Baltim). 2024;103:e39476. 10.1097/MD.0000000000039476 PMC11404896 39287304

[5] Park J Cheon JH Lee KM. Early infliximab trough levels predict the long-term efficacy of infliximab in a randomized controlled trial in patients with active Crohn’s disease comparing, between CT-P13 and originator infliximab. Gut Liver. 2023;17:430–40. 35975641 10.5009/gnl220005 PMC10191793

[6] Jørgensen KK Goll GL Sexton J. Efficacy and safety of CT-P13 in inflammatory bowel disease after switching from originator infliximab: exploratory analyses from the NOR-SWITCH main and extension trials. BioDrugs. 2020;34:681–94. 32965617 10.1007/s40259-020-00438-7 PMC7519917

[7] Papamichael K Lin S Moore M Papaioannou G Sattler L Cheifetz AS. Infliximab in inflammatory bowel disease. Ther Adv Chronic Dis. 2019;10:2040622319838443. 30937157 10.1177/2040622319838443 PMC6435871

[8] Farkas K Rutka M Ferenci T. Infliximab biosimilar CT-P13 therapy is effective and safe in maintaining remission in Crohn’s disease and ulcerative colitis – experiences from a single center. Expert Opin Biol Ther. 2017;2017:1–8. 10.1080/14712598.2017.1363885 28819991

[9] Turner D Ricciuto A Lewis A. STRIDE-II: an update on the selecting therapeutic targets in Inflammatory Bowel Disease (STRIDE) Initiative of the International Organization for the Study of IBD (IOIBD): determining therapeutic goals for treat-to-target strategies in IBD. Gastroenterology. 2021;160:1570–83. 33359090 10.1053/j.gastro.2020.12.031

[10] Inflammatory Bowel Disease Group, Chinese Society of Gastro-enterology, Chinese Medical Association. Chinese consensus on diagnosis and treatment in inflammatory bowel disease (2018, Beijing). J Dig Dis. 2021;22:298–317. 33905603 10.1111/1751-2980.12994

[11] Patel D. Sample size estimation in clinical trials. Natl J Community Med. 2024;15:503–8.

[12] D’Haens G Sandborn WJ Feagan BG. A review of activity indices and efficacy end points for clinical trials of medical therapy in adults with ulcerative colitis. Gastroenterology. 2007;132:763–86. 17258735 10.1053/j.gastro.2006.12.038

[13] Travis SPL Schnell D Krzeski P. Developing an instrument to assess the endoscopic severity of ulcerative colitis: the Ulcerative Colitis Endoscopic Index of Severity (UCEIS). Gut. 2012;61:535–42. 21997563 10.1136/gutjnl-2011-300486 PMC3292713

[14] Best WR Becktel JM Singleton JW Kern F. Development of a Crohn’s disease activity index. National Cooperative Crohn’s Disease Study. Gastroenterology. 1976;70:439–44. 1248701

[15] Daperno M D’Haens G Van Assche G. Development and validation of a new, simplified endoscopic activity score for Crohn’s disease: the SES-CD. Gastrointest Endosc. 2004;60:505–12. 15472670 10.1016/s0016-5107(04)01878-4

[16] Satsangi J Silverberg MS Vermeire S Colombel JF. The Montreal classification of inflammatory bowel disease: controversies, consensus, and implications. Gut. 2006;55:749–53. 16698746 10.1136/gut.2005.082909 PMC1856208

[17] Kumar P Vuyyuru SK Kante B. Efficacy and safety of biosimilar versus originator infliximab in patients with inflammatory bowel disease: a real-world cohort analysis. Indian J Gastroenterol. 2022;41:446–55. 36378484 10.1007/s12664-022-01252-5

[18] Kim NH Lee JH Hong SN. Long‐term efficacy and safety of CT‐P13, a biosimilar of infliximab, in patients with inflammatory bowel disease: a retrospective multicenter study. J Gastroenterol Hepatol. 2019;34:1523–32. 30828891 10.1111/jgh.14645

[19] Gonczi L Gecse KB Vegh Z. Long-term efficacy, safety, and immunogenicity of biosimilar infliximab after one year in a prospective nationwide cohort: inflammatory bowel diseases. Inflamm Bowel Dis. 2017;23:1908–15. 28922253 10.1097/MIB.0000000000001237

[20] Neurath MF Vieth M. Different levels of healing in inflammatory bowel diseases: mucosal, histological, transmural, barrier and complete healing. Gut. 2023;72:2164–83. 37640443 10.1136/gutjnl-2023-329964

[21] Sands BE Danese S Chapman JC. Mucosal and transmural healing and long-term outcomes in Crohn’s disease. Inflamm Bowel Dis. 2025;31:857–77. 39083264 10.1093/ibd/izae159 PMC11879194

[22] Shah SC Colombel JF Sands BE Narula N. Mucosal healing is associated with improved long-term outcomes of patients with ulcerative colitis: a systematic review and meta-analysis. Clin Gastroenterol Hepatol. 2016;14:1245–55.e8. 26829025 10.1016/j.cgh.2016.01.015

[23] Boal Carvalho P Cotter J. Mucosal healing in ulcerative colitis: a comprehensive review. Drugs. 2017;77:159–73. 28078646 10.1007/s40265-016-0676-y

[24] Bertani L Fornai M Fornili M. Serum oncostatin M at baseline predicts mucosal healing in Crohn’s disease patients treated with infliximab. Aliment Pharmacol Ther. 2020;52:284–91. 32506635 10.1111/apt.15870

[25] Nakagawa T Kobayashi T Nishikawa K. Infliximab biosimilar CT-P13 is interchangeable with its originator for patients with inflammatory bowel disease in real world practice. Intest Res. 2019;17:504–15. 31422647 10.5217/ir.2019.00030 PMC6821950

[26] Gecse KB Lovász BD Farkas K. Efficacy and safety of the biosimilar infliximab CT-P13 treatment in inflammatory bowel diseases: a prospective, multicentre, Nationwide Cohort. J Crohns Colitis. 2016;10:133–40. 26661272 10.1093/ecco-jcc/jjv220

[27] Guerra Veloz MF Argüelles-Arias F Castro Laria L. Loss of efficacy and safety of the switch from infliximab original to infliximab biosimilar (CT-P13) in patients with inflammatory bowel disease. World J Gastroenterol. 2018;24:5288–96. 30581277 10.3748/wjg.v24.i46.5288 PMC6295832

